# Maternal High Fat Diet Anticipates the AD-like Phenotype in 3xTg-AD Mice by Epigenetic Dysregulation of Aβ Metabolism

**DOI:** 10.3390/cells12020220

**Published:** 2023-01-04

**Authors:** Francesca Natale, Matteo Spinelli, Marco Rinaudo, Sara Cocco, Ida Nifo Sarrapochiello, Salvatore Fusco, Claudio Grassi

**Affiliations:** 1 Department of Neuroscience, Università Cattolica del Sacro Cuore, 00168 Rome, Italy; 2 Fondazione Policlinico Universitario Agostino Gemelli IRCCS, 00168 Rome, Italy

**Keywords:** Alzheimer’s disease, Bace1, epigenetics, maternal HFD, Insulin degrading enzyme, NF-kB, STAT3, amyloid-β-protein

## Abstract

Maternal overnutrition has been reported to affect brain plasticity of the offspring by altering gene expression, regulating both synaptic plasticity and adult neurogenesis. However, whether perinatal metabolic stress may influence the accumulation of misfolded proteins and the development of neurodegeneration remains to be clarified. We investigated the impact of maternal high fat diet (HFD) in an experimental model of Alzheimer’s disease (AD). The 3xTg-AD mice born to overfed mothers showed an impairment of synaptic plasticity and cognitive deficits earlier than controls. Maternal HFD also altered the expression of genes regulating amyloid-β-protein (Aβ) metabolism (i.e., Bace1, Ern1, Ide and Nicastrin) and enhanced Aβ deposition in the hippocampus. Finally, we found an epigenetic derangement and an aberrant recruitment of transcription factors NF-kB and STAT3 and chromatin remodeler HDAC2 on the regulatory sequences of the same genes. Collectively, our data indicate that early life metabolic stress worsens the AD phenotype via epigenetic alteration of genes regulating Aβ synthesis and clearance.

## 1. Introduction

Alzheimer’s disease (AD) is an irreversible neurodegenerative disorder that affects brain structure and activity, and progressively leads to the decline of cognitive function in the elderly population [1]. The AD brain is characterized by the dysregulation of multiple pathways causing neuroinflammation, oxidative stress, mitochondrial and protein dyshomeostasis, which appear as an alteration of synaptic functions and the development of molecular hallmarks including deposition of both amyloid-β-protein (Aβ) and phosphorylated microtubule-associated protein Tau (i.e., neurofibrillary tangles, NFTs) [2,3]. Linkage and genome-wide association studies have mapped several regions in human genome associated with AD risk [4]: specific genes, such as APOE and BACE1/2, are thought to be prime candidates to confer risk because of their role in pathways involving Aβ biosynthesis and deposition [5]. Interestingly, non-genetic factors such as diet and nutrient-related signals, may also be involved in the pathogenesis and progression of AD. A new view is thus arising according to which poor lifestyle habits (e.g., unhealthy diets) may affect brain health, and constitute a significant risk factor for AD [6]. We showed that a high fat diet (HFD) caused alterations of synaptic function, adult neurogenesis, learning and memory resembling AD-like phenotype [7,8]. We also demonstrated that maternal HFD multigenerationally caused a derangement of cognitive functions by epigenetically inhibiting genes regulating brain plasticity [9]. Indeed, early life stress may be a long lasting influence gene expression via a plethora of mechanisms, including epigenetic changes such as the post-translational modifications of histone proteins and DNA methylation [10]. However, whether perinatal metabolic stress may persistently affect the expression of genes regulating Aβ/Tau metabolism and influence the development of neurodegeneration remain largely unknown. Here, we demonstrate that maternal HFD caused the early onset of AD-like phenotype in 3xTg-AD mice by anticipating Aβ deposition, synaptic plasticity impairment and cognitive deficits. Moreover, we find NF-kB- and STAT3-driving epigenetic derangement of regulatory sequences controlling the expression of Bace1, Ern1, Ide and Nicastrin genes in the hippocampus of the offspring born to overfed 3xTg-AD female mice.

## 2. Materials and Methods

### 2.1. Animals

Female 3xTg-AD mice (30 days old), derived from the Animal Facility of Catholic University, were randomly assigned to two feeding regimens: (i) SD (control diet) and (ii) HFD until they were ready for mating, and were weighed weekly. Female mice (F0) were paired for breeding at the end of the fourth week of dietary regimen as previously published [11]. F0 pregnant females were fed with HFD until the second week of lactation. Male mice (including the F1_SD_ and F1_HFD_ offspring) were always fed with standard chow. The same male mouse was paired, at different times, with both a F0_SD_ and a F0_HFD_ female mouse and was exposed to HFD only during this time lapse. A maximum of two male offspring were taken from each litter for each experimental set to remove any litter effects.

### 2.2. Diet and Housing Conditions

F0 mice were fed with SD (18.5% proteins; 46% carbohydrates, namely 42% starch, 4% sucrose; 3% fats; 6.55% fat caloric content; cat. num. 4RF21) or HFD (23% proteins; 42% carbohydrates, namely 28% starch, 9% sucrose, 5% maltodextrin; 34% fats; 60% fat caloric content; cat. num. PF4051/D) for 6 weeks. Diets, which are balanced for fiber content (5%), were purchased from Mucedola (Italy). Chows were stored at 4°C and, in cages, diet was replaced weekly to prevent degradation. Mice were housed in cages with the following measures: 25 cm × 20 cm × 15 cm, under a 12-h light–dark cycle at room temperature (RT, 19–22 °C), fed with their respective diets and received water ad libitum. Weight and food consumption were weekly monitored.

### 2.3. Behavioral Experiments

Behavioral tests were carried out from 9 a.m. to 4 p.m. and data were analyzed in blind using an automated video tracking system (Any-Maze™). Recognition memory was evaluated by novel object recognition (NOR) test. On first day, animals were familiarized for 10 min to the test arena (45 × 45 cm). On second day (training session), they were allowed to explore two identical objects placed symmetrically in the arena for 10 min. On third day (test session), a new object replaced one of the old objects. Animals were allowed to explore for 10 min and preference index, calculated as the ratio between time spent exploring the novel object and time spent exploring both objects, was used to measure recognition memory. For both training and test, animals exploring the objects for a total time of less than 20 s were excluded from the analysis. Spatial learning and memory were assessed using the Morris water maze (MWM) test. A circular plastic pool (127 cm in diameter) filled with water colored with nontoxic white paint to obscure the location of a hidden platform was used as experimental apparatus. The pool was ideally separated into four equal quadrants (NE, corresponding to the target quadrant, SE, NW, and SW) and the platform (10 × 10 cm) was placed at the center of the target quadrant. Visual cues were placed on the walls around the pool to orient the mice. Animals were trained for four days, six times a day, and the probe test was administered 24 h after the last training day. Starting positions were varied daily and both latencies and distances to reach the platform were recorded. In the probe test, the platform was removed, and time spent in each quadrant was measured (60 s of test duration). All experimental groups did not show any statistical difference in terms of average swimming speed or thygmotactic behavior during the tests.

### 2.4. Ex Vivo Electrophysiology on Hippocampal Slices

Field excitatory postsynaptic potentials (fEPSPs) were elicited in the CA1 area of hippocampus by placing a bipolar concentric stimulating electrode (FHC) in the Schaffer collateral pathway. The electrode was connected to a constant current isolated stimulator (Digitimer). A low impedance glass pipette (1–2 MΩ) was filled with ACSF and placed immediately below the CA1 stratum pyramidale. Recordings were performed in current clamp I = 0 mode, using a Multiclamp 700A/Digidata 1440A system (Molecular Devices). First, the input-output relationship was constructed and the stimulus intensity resulting in 30% of maximal response amplitude was found. After achieving a stable baseline response, LTP was induced by using the high-frequency stimulation protocol (1 train of stimuli at 100 Hz, lasting 500 ms, repeated four times with an inter-train interval of 20 s). After LTP induction, fEPSP amplitude and slope were monitored for at least 60 min. Data were analyzed as previously described [7].

### 2.5. Real-Time PCR

RT-PCR experiments were performed using RT2 SYBR^®^ Green ROX qPCR Mastermix (Qiagen) on AB7500 instrument (Life Technologies, Carlsbad, CA, USA) according to the manufacturer’s instructions. The thermal cycling program consisted in a pre-incubation step of 94 °C for 10 min, followed by 40 cycles of denaturation (94 °C, 15 s), annealing (55 °C, 30 s), and elongation (72 °C, 20 s). For each RT-PCR experiment, only single products had been amplified, as confirmed by melting curves subsequently generated (94 °C for 15 s, 50 °C for 30 s, slow heating to 94 °C in increments of 0.5 °C). For PCR array experiments, the mRNA levels of 89 genes of interests plus 5 housekeeping genes were simultaneously examined through an RT2 Profiler Custom PCR Array (PAMM-057Z) in 96-well plates according to manufacturer’s protocol (Qiagen). Each experiment (one 96 well plate) included 40 ng of total extracted RNA and the negative controls (no template, no reverse transcription,). All samples (n = 4 biological replicates per condition) were randomly selected and analyzed in triplicate, and data were normalized to glyceraldehyde 3-phosphate dehydrogenase levels using the ΔΔCt method. Results are shown in Supplementary Table S1.

### 2.6. ELISA Assay

Hippocampal Aβ 1–42 concentration was measured by using the Amyloid beta 42 Human ELISA Kit, Ultrasensitive, Thermofisher Scientific. The assays were performed according to the manufacturer’s instructions.

### 2.7. Western Blotting

Tissues (n = 6 biological replicates per condition) were lysed in ice-cold lysis buffer (NaCl 150 mM, Tris-HCl 50 mM pH 7.4, EDTA 2 mM) containing 1% Triton X-100, 0.1% SDS, 1× protease inhibitor cocktail (Sigma-Aldrich, St. Louis, MO, USA), 1 mM sodium orthovanadate (Sigma-Aldrich) and 1 mM sodium fluoride (Sigma-Aldrich) as previously described [12]. After a 10 min incubation on ice at 4 °C with occasional vortexing, homogenates were sonicated on ice for 5 min and centrifuged at 13,000× *g* for 15 min at 4 °C. Protein content in the supernatant was quantified through the Bradford assay (DC Protein Assay; Bio-Rad). 40–60 μg of proteins from total lysates were diluted in Laemmli buffer, boiled for 5 min at 100 °C, and resolved using a SDS-PAGE polyacrylamide gel. Each nitrocellulose membrane has been cut in two parts and hybridized with different antibodies against proteins of different molecular weight. When necessary (e.g., target with similar molecular weight and same secondary antibody), membranes have been stripped before re-hybridization at most once. The primary antibodies (1 μg/mL, diluted in TBS-Tween20, 3% non-fat dried milk) were incubated overnight at 4 °C on a plate shaker and revealed with horseradish peroxidase-conjugated secondary antibodies (1:5000 in TBS-Tween20, Cell Signaling Technology Inc., Danvers, MA, USA). Single protein expression was evaluated using UVItec Cambridge Alliance Software. Images shown were cropped for presentation with no manipulations. All uncropped blots are included in supplementary files. Antibodies are available in Supplementary Table S2.

### 2.8. Chromatin Immunoprecipitation

Chromatin immunoprecipitation (ChIP) assays were performed as previously described [13]. Hippocampi (n = 6 biological replicates per condition) were resuspended in 200 μL lysis buffer containing 1% SDS, 50 mM Tris-HCl pH 8.0, and 10 mM EDTA and sonicated on ice with six 10 s pulses with a 20 s interpulse interval. Sample debris was removed by centrifugation and supernatants were precleared with protein-G Sepharose 4B beads (Sigma-Aldrich) for 1 h at 4 °C. 2 μg of specific antibody or control IgG were added overnight at 4 °C. Immune complexes were collected by incubation with protein-G Sepharose 4B beads for 2 h at 4 °C. After seven sequential washes, immune complexes were eluted from beads by vortexing in elution buffer (1% SDS and NaHCO_3_ 0.1 M; pH 8.0). NaCl was added (final concentration 0.33 M), and cross-linking was reversed by incubation overnight at 65 °C. DNA fragments were purified by using the PCR DNA fragments purification kit (Geneaid). The primer sequences are shown in Supplementary Table S3. PCR conditions and cycle numbers were empirically determined, and each PCR reaction was performed in triplicate. Data are expressed as percentage of input calculated by the “Adjusted input value” method according to the manufacturer’s instructions (ThermoFisher Scientific ChIP Analysis). To calculate the adjusted input the Ct value of input was subtracted by 6.644 (i.e., log2 of 100). Next, the percent input of samples was calculated using the formula: 100 × 2^(Adjusted input—Ct(ChIP). The percent input of IgG samples was calculated using the formula 100 × 2^(Adjusted input—Ct(IgG).

### 2.9. Statistical Analysis

All statistical analyses, including sample size calculation, were performed using the software SigmaPlot 14.0. Sample sizes were estimated with adequate power (0.8) following results of prior pilot datasets or studies based on similar methods or paradigms, including our own. Prior to statistical tests, equal variance and normality (Shapiro-Wilk test) were assessed. The statistical tests and post hoc comparisons used are reported in the corresponding figure legends for each experiment. The level of significance was set at 0.05 and all statistical tests were two-tailed. Results are expressed as mean ± SEM.

## 3. Results

### 3.1. Maternal HFD Anticipates LTP Impairment and Memory Deficits in 3xTg-AD Mice

**Figure 1:**
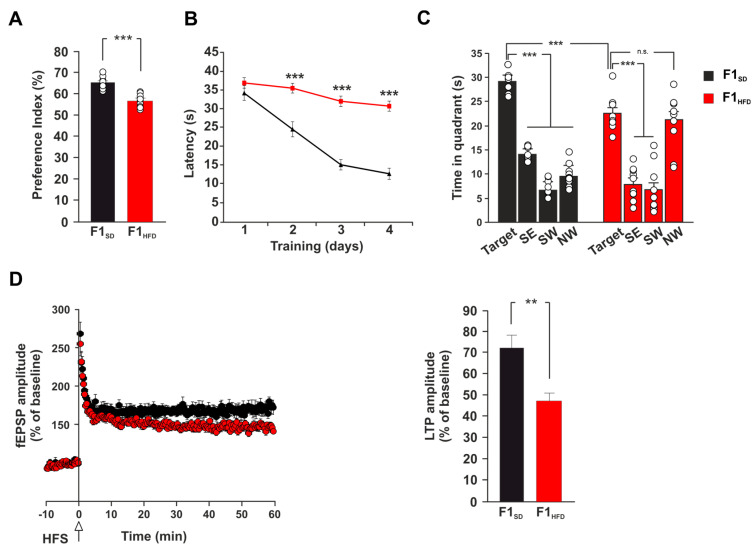
Maternal HFD anticipates the onset of learning and memory deficits in 3xTg-AD mice. (**A**) Preference for the novel object of offsprings derived from female 3xTg-AD mice fed SD or HFD (F1_SD_ or F1_HFD_, respectively) (n = 10 for each group; statistics by unpaired Student’s *t*-test). (**B**) Latency to reach the platform in MWM test (n = 10 for each group; statistics by unpaired Student’s *t*-test). (**C**) Time spent in the four quadrants during MWM probe test. NE is the target quadrant (n = 10 for each group; statistics by unpaired Student’s t test for time in the target quadrant and one-way ANOVA and Bonferroni post hoc for time in all quadrants). (**D**) Time course (left) of LTP at CA3-CA1 synapses in hippocampal slices of F1_SD_ and F1_HFD_ mice. Results are expressed as percentages of baseline EPSC amplitude (=100%). On the right, mean LTP values during the last 5 min (n = 10 for F1_HFD_ and n = 11 for F1_SD_; statistics by unpaired Student’s *t*-test). Data are expressed as mean ± SEM. ** *p* < 0.01; *** *p* < 0.001; n.s. not significant.

Early life stress may induce long-term neurobiological modifications (e.g., the alteration of adult neurogenesis, dendritic spine formation and synaptic activity) affecting neuroplasticity and brain health in the adulthood [14,15,16]. Maternal overnutrition has been reported to affect brain plasticity and cognitive function of the offspring [17,18,19]. However, it is unknown whether metabolic stress in the early stage of life can also persistently influence the progression of neurodegeneration in an experimental model of AD. To test this hypothesis, we used 3xTg-AD transgenic mice, which express three major genes associated with familial AD, as well as the corresponding behavioral and neuropathological changes that are observed in the human form [20]. In this transgenic model the disease progression is well characterized. The earliest cognitive impairment appears at six months and correlates with the accumulation of intraneuronal Aβ in the hippocampus. These mice progressively exhibit impaired hippocampal synaptic dysfunction (including LTP deficits) and cognitive deficits in an age-related manner [21,22]. We fed female 3xTg-AD mice (F0) with control diet (SD) or HFD for four weeks before mating, during the pregnancy, and until the second week of lactation. The offspring (F1_SD_ or F1_HFD_, respectively) were always fed with SD since weaning and were analyzed at four months of age to investigate if maternal overnutrition modified the onset of behavioral and functional changes in these mice. We evaluated the hippocampus-dependent learning and memory of the offspring by novel object recognition (NOR) test and Morris water maze (MWM) task at a stage when 3xTg-AD mice exhibit no behavioral alterations. In the NOR test, F1_HFD_ 3xTg-AD mice showed less preference for the novel object than controls (56.8 ± 0.9% vs. 65.1 ± 0.9%; n = 10; P = 4.99 × 10^−6^; Figure 1A).

Moreover, 3xTg-AD animals derived from overfed mothers took longer time to reach the hidden platform during the training phase of MWM test (34.9 ± 0.8 vs. 24.5 ± 1.5 s for day 2; 32.1 ± 1.1 vs. 15.1 ± 1.7 s for day 3; 30.1 ± 1.1 vs. 12.7 ± 1.6 s for day 4; n = 10; *p* < 0.001 for each day; Figure 1B). Accordingly, they also spent less time than controls in the target quadrant and did not distinguish the target quadrant from all other ones during the probe test (time in the target quadrant: 22.9 ± 1.2 vs. 29.1 ± 0.7 s, *p* = 1.37 × 10^−4^; time in the four quadrants: North–East (NE) vs. North–West (NW), *p* = 0.489 for F1_HFD_ 3xTg-AD mice; n = 10; Figure 1C). Therefore, the analysis of long-term potentiation (LTP) at the CA3–CA1 hippocampal synapses in brain slices derived from F1_SD_ and F1_HFD_ 3xTg-AD mice revealed a significant deficit of synaptic plasticity in the offspring of overfed mothers (field excitatory postsynaptic potential (fEPSP) amplitude potentiation: 47.4 ± 4.7% vs. 71.8 ± 7.1%, *p* = 0.0015; n = 10 for F1_HFD_ and n = 11 for F1_SD_; Figure 1D). Collectively, our in vivo and ex vivo experiments indicated that metabolic stress occurring in the early phase of brain development accelerated the impairment of synaptic plasticity, learning and memory in 3xTg-AD mice and anticipated the onset of AD-like phenotype.

### 3.2. Maternal HFD Alters the Expression of AD-Related Genes and Enhances Aβ Deposition in the Hippocampus of the Offspring

**Figure 2:**
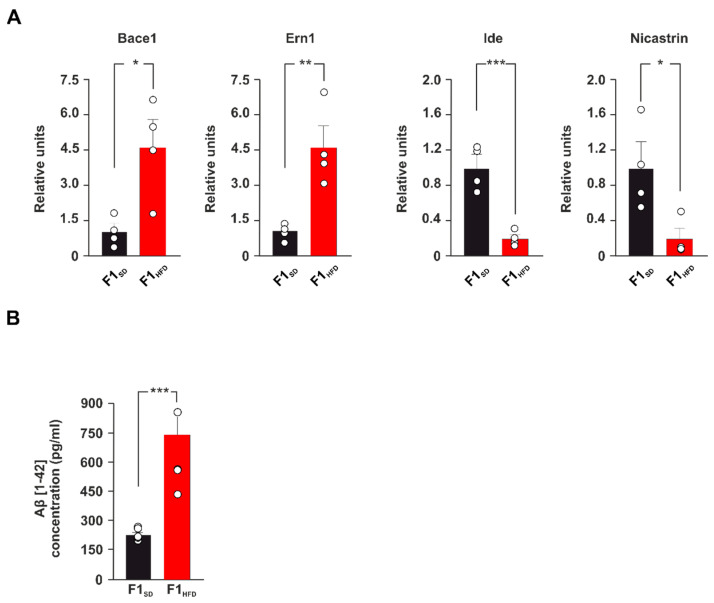
Maternal HFD induces alteration of AD-related gene expression and increase of Aβ deposition in the hippocampus. (**A**) Up-down fold expression changes of genes significantly altered in the hippocampus of F1_SD_ or F1_HFD_ 3xTg-AD mice (n = 4 mice per experimental group). Real-time (RT)-PCR was performed in triplicate. The bar graphs show genes with fold change ≥2 and *p* value < 0.05. The full list of genes and fold expression changes is shown in Supplementary Table S1. (**B**) Aβ 1-42 levels in the hippocampus of F1_SD_ and F1_HFD_ mice. ELISA assay was performed in duplicate (n = 6 mice per group; statistics by unpaired Student’s *t*-test). Data are expressed as mean ± SEM. * *p* < 0.05; ** *p* < 0.01; *** *p* < 0.001.

To gain insight into the mechanisms underlying the worsening of the AD phenotype in the 3xTg-AD mice born to HFD mothers, we studied the expression of a pattern of AD-related genes in hippocampal lysates of F1_SD_ and F1_HFD_ 3xTg-AD animals. Real-time PCR array revealed either upregulation or downregulation of several genes potentially regulating the development of neurodegeneration in the offspring of overfed mothers (Supplementary Table S1). In particular, the expression of genes coding for the enzymes BACE1, IRE1 (i.e., Ern1), IDE and Nicastrin, which control Aβ metabolism, was altered in the hippocampus of F1_HFD_ 3xTg-AD mice compared to controls (fold change: +4.76 for Bace1, *p* = 0.0158; +4.57 for Ern1, *p* = 0.0059; −4.95 for Ide, *p* = 0.0009; −5.16 for Nicastrin, *p* = 0.0235; n = 4; Figure 2A).

Remarkably, the beta-secretasi 1 (BACE1) cleaves APP protein and it is an essential enzyme for the generation of β-amyloid (Aβ) [23,24]. Conversely, Nicastrin is a regulator of Neprilysin, which together with Insulin Degrading Enzyme (IDE) are the main enzymes contributing to the clearance of Aβ [25,26]. To evaluate the impact of dysregulated expression of these enzymes on Aβ deposition, we analyzed the Aβ1-42 levels in the hippocampus of F1_HFD_ 3xTg-AD mice. ELISA assay showed a significant increase of Aβ levels at early stage of disease in 3xTg-AD mice born to overfed mothers compared to controls (738.77 ± 116.37 vs. 222.03 ± 12.83 pg/mL; *p* = 6.73 × 10^−4^; n = 6; Figure 2B). Collectively, our findings revealed that maternal HFD induced aberrant expression of genes regulating neurodegeneration and AD-like phenotype and increased Aβ deposition in the hippocampus of 3xTg-AD mice.

### 3.3. Maternal Overnutrition Induces Hyper-Activation of Transcription Factors NF-kB and STAT3

**Figure 3:**
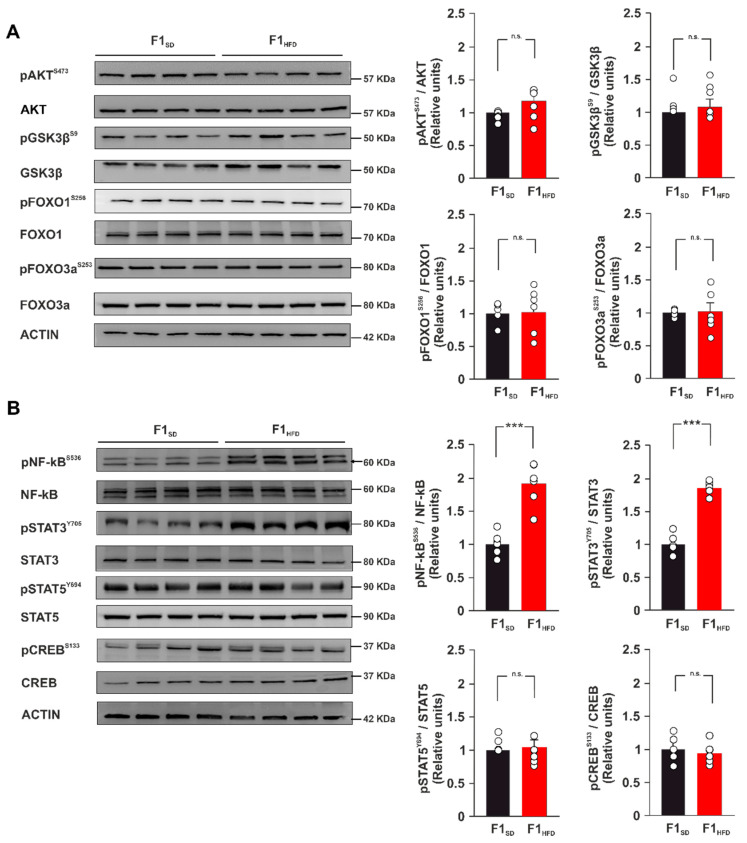
Maternal HFD increases the phosphorylation levels of both NF-kB and STAT3. (**A**) Immunoblots and bar graphs showing the expression and phosphorylation levels of brain insulin resistance protein markers (AKT, GSK3β, FOXO1, and FOXO3a) in F1_SD_ and F1_HFD_ 3xTg-AD mice (n = 6 mice per group; statistics by unpaired Student’s *t*-test). (**B**) Immunoblots and bar graphs showing the expression and phosphorylation levels of transcription factors NF-kB, STAT3, STAT5, and CREB (n = 6 mice per group; statistics by unpaired Student’s *t*-test). Data are expressed as mean ± SEM. *** *p* < 0.001; n.s., not significant.

HFD has been shown to induce in the hippocampus changes of insulin receptor-downstream effectors resembling the alteration of insulin signaling observed in other tissues [7]. Moreover, molecular markers of brain insulin resistance have been found in 3xTg-AD hippocampal tissues [27,28]. To understand the molecular cascades involved in the dysregulated expression of AD-related genes, we analyzed both the expression and the activation of the main insulin downstream signaling proteins in the hippocampus of F1_HFD_ 3xTg-AD mice compared to controls. The levels of protein-kinase B (PKB/AKT), Glycogen synthase kinase-3 beta (GSK3β), transcription factors Forkhead box O1 (FoxO1) and Forkhead box O3a (FoxO3a), and their phosphorylation were not significantly altered in the offspring of overfed mothers (*p* > 0.05 for all comparisons; n = 6; Figure 3A). We also investigated the activation state of several nutrient-responsive transcription factors including cAMP response element-binding protein (CREB), Nuclear Factor kappa B p65 (NF-kB) and Signal transducer and activator of transcription 3 (STAT3), which were identified by the bioinformatic analysis of the RT-PCR array results as potential regulators of Bace1, Ern1, Ide and Nicastrin genes. Immunoblot analysis detected hyperphosphorylation of both NF-kB and STAT3 in the hippocampi of F1_HFD_ 3xTg-AD mice (pNF-kB^Ser536^: +92.1%, *p* = 0.0001; pSTAT3^Tyr705^: +96.5%, *p* = 2.53 × 10^−7^; n = 6; Figure 3B). Our data revealed that maternal overnutrition enhanced the activation of nutrient- and AD-related transcription factors NF-kB and STAT3 in the hippocampus of adult 3xTg-AD mice.

### 3.4. Maternal HFD Epigenetically Dysregulates the Promoters of Genes Driving APP Metabolism in the Hippocampus of 3xTg-AD Mice

**Figure 4:**
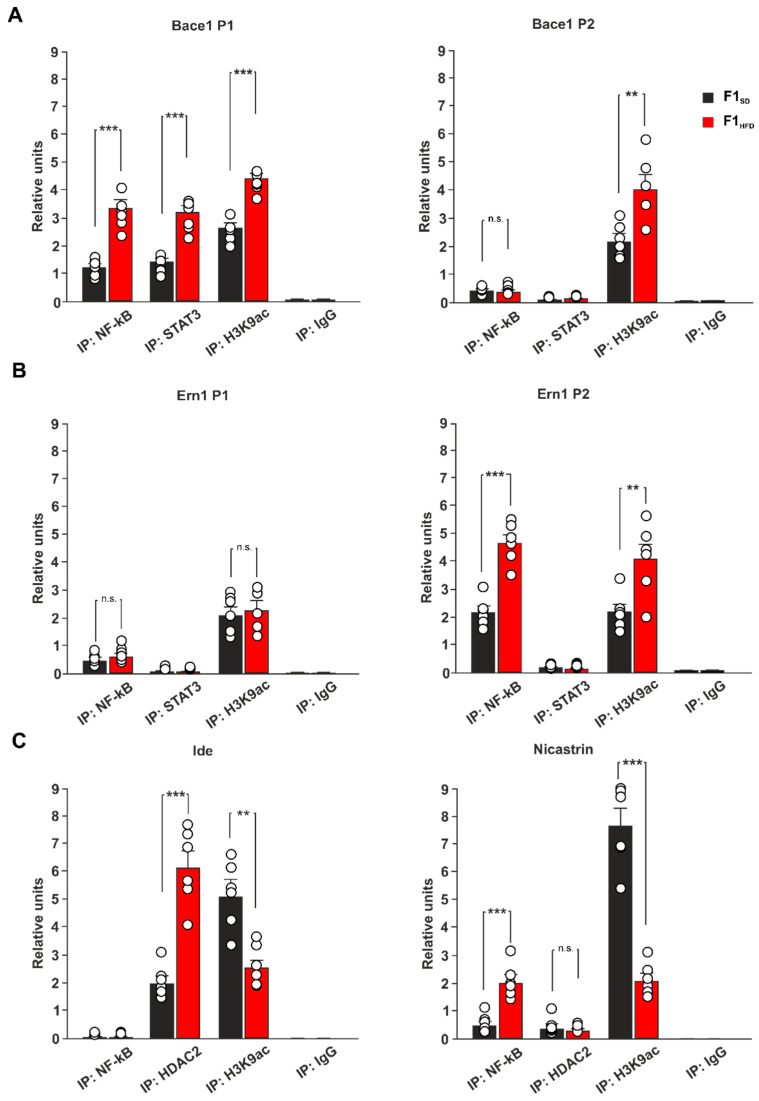
Ancestor HFD induced epigenetic alteration in the promoters of genes involved in AD onset and pathogenesis. (**A**) ChIP assays of NF-kB, STAT3 and H3K9ac on two different promoters of Bace1 gene in F1_SD_ and F1_HFD_ 3xTg-AD mice (n = 6; statistics by one-way ANOVA and Bonferroni post hoc). (**B**) ChIP assays of NF-kB, STAT3 and H3K9ac on two different promoters of Ern1 gene in F1_SD_ and F1_HFD_ 3xTg-AD mice (n = 6; statistics by one-way ANOVA and Bonferroni post hoc). (**C**) ChIP assays of NF-kB, STAT3 and H3K9ac on the promoters of Ide and Nicastrin genes in F1_SD_ and F1_HFD_ 3xTg-AD mice (n = 6; statistics by one-way ANOVA and Bonferroni post hoc). IgG are used as negative controls. Real-time analysis was performed in triplicate. Data are expressed as mean ± SEM. ** *p* < 0.01; *** *p* < 0.001; n.s. not significant.

Early-life stress affects brain plasticity in adulthood and increases vulnerability to neuropsychological disorders [29]. Moreover, increasing evidence suggests that epigenetic changes can mediate the impact of environmental stress on AD onset [30]. In addition, we showed that maternal HFD induced an impairment of cognitive functions in the descendants by epigenetically affecting the expression of brain plasticity-related genes in the hippocampus [9]. To investigate the role of NF-kB, STAT3 and epigenetic modifications in the HFD-dependent alteration of AD-related gene expression, we analyzed the levels of both transcription factors and transcriptional activity marker histone H3 lysine 9 acetylation (H3K9ac) on the promoters of Bace1 and Ern1 genes. We found elevated binding of both NF-kB and STAT3, and higher levels of H3K9ac on the regulatory sequences of Bace1 in the hippocampi of F1_HFD_ 3xTg-AD mice (P1: +166.1% for NF-kB, *p* = 2.63 × 10^−5^, +146.7% for STAT3, *p* = 3.34 × 10^−5^, +75.2% for H3K9ac, *p* = 1.19 × 10^−5^; P2: +87.4% for H3K9ac, *p* = 0.0056; n = 6; Figure 4A).

In addition, both increased recruitment of NF-kB and elevated levels of histone acetylation were also evident on the promoter of Ern1 gene by chromatin immunoprecipitation (ChIP) experiments (P2: +115.8% for NF-kB, *p* = 4.75 × 10^−5^; +87.3% for H3K9ac, *p* = 0.0091; n = 6; Figure 4B). Conversely, the regulatory sequences of downregulated Ide and Nicastrin showed enhanced binding of epigenetic repressor histone deacetylase 2 (HDAC2) and reduced levels of H3K9ac in the hippocampi of HFD offspring (Ide: +203.4% for HDAC2, *p* = 5.26 × 10^−5^, −49.9% for H3K9ac, *p* = 0.0013; Nicastrin: −72.7% for H3K9ac, *p* = 6.85 × 10^−6^; n = 6; Figure 4C), indicating that epigenetic derangement of AD-related gene promoters may be involved in the maternal HFD-dependent worsening of AD phenotype. No binding was detected for transcription factor NF-kB on the promoter of Ide, whereas higher recruitment occurred on the promoter of Nicastrin in the hippocampi of mice derived from overfed mothers (+310.2% for F1_HFD_ 3xTg-AD mice, *p* = 0.0003; n = 6; Figure 4B). Collectively, our results reveal that maternal overnutrition can accelerate AD progression in 3xTg-AD mice via dysregulation of NF-kB, STAT3 and HDAC2 recruitment on the regulatory sequences of genes regulating APP metabolism.

## 4. Discussion

AD is a neurodegenerative disorder that progressively leads to the impairment of cognitive function, and it represents the most common cause of dementia in the elderly population [31]. It is a multifactorial disease characterized by aberrant synaptic function due to different pathogenic mechanisms including the development of neuroinflammation and oxidative stress, the reduction of neurotrophic factor levels, the alteration of mitochondrial activity, and others still not fully identified [32]. The triggering of a vicious cycle among all these factors contributes to the development of protein dyshomeostasis and AD molecular hallmarks, such as the deposition of both amyloid-β-protein (Aβ) and phosphorylated microtubule-associated protein Tau in the brain [33]. Emerging evidence suggests that environmental stress, such as an alteration of metabolic homeostasis, may accelerate brain aging and increase the risk of dementia [34,35]. More importantly, AD onset and progression are associated with epigenetic changes of key AD-related genes that play a critical role in the development of neurodegeneration [30]. It is now clearly emerging that epigenetic modifications represent the molecular expression of the interaction between environmental factors and genome, and have a pivotal role in mediating the susceptibility to various diseases [36]. However, whether maternal HFD may epigenetically modulate the AD phenotype in the offspring remains largely unexplored. Here, we demonstrate that early life metabolic stress induces an anticipation of synaptic impairment and cognitive deficits in 3xTg-AD mice (Figure 1). Maternal overnutrition has been shown to increase Aβ pathology and to worsen memory deficits in experimental models of AD [37,38]. We detected an aberrant expression of genes coding enzymes regulating Aβ synthesis and clearance (i.e., Bace1, Ern1, Ide, Nicastrin) and higher levels of Aβ1-42 in the hippocampi of F1_HFD_ 3xTg-AD mice (Figure 2). BACE1 is a protease enzyme that cleaves the transmembrane APP and contributes to generate Aβ species [24]. It has been also demonstrated that overnutrition-related signals upregulate the expression of Bace1 [39]. Accordingly, IDE can be finely modulated by metabolic stimuli, and it has been proposed as a key molecular link between AD and insulin resistance [40,41]. It has been demonstrated that HFD may induce hippocampal insulin resistance by altering the activation of insulin receptor downstream effectors and transcription factors [27]. However, mice born to overfed mothers did not show molecular hallmarks of brain insulin resistance in their hippocampi. Instead, the immunoblot analysis of nutrient-related transcription factors revealed hyper-activation of both NF-kB and STAT3 in the hippocampi of F1_HFD_ offspring (Figure 3). Transcription factor NF-kB is required for hippocampal long-term synaptic plasticity and memory formation [42], but it has been also demonstrated its critical role in the neuroinflammatory responses triggering neurodegeneration [43,44]. Moreover, it has been reported that Aβ impairs synaptic plasticity by enhancing NF-kB activity [45], but also that NF-kB phosphorylation increases both the expression of protease Bace1 and the generation of beta-amyloid peptides [46], describing a sort of vicious cycle surrounding the transcription factor and promoting the Aβ-mediated synaptotoxicity. The inhibition of STAT3 transcriptional activity has also been described to counteract AD phenotype [47,48]. In addition, STAT3 has been shown to regulate Bace1 expression [49] and to be regulated by HFD-related signals in the hippocampus [50]. The bioinformatic analysis revealed several putative NF-kB and STAT3 binding regions on the promoters of AD-related genes whose expression was altered by maternal overnutrition. ChIP assays confirmed an epigenetic derangement on the regulatory sequences of Bace1, and Ern1 genes, where we found higher recruitment of both transcription factors and increased levels of histone 3 acetylation (Figure 4). Conversely, the promoters of downregulated Ide and Nicastrin genes showed the enhanced binding of transcriptional repressor HDAC2 and lower levels of H3K9ac. Increased H3 acetylation at the promoter of the BACE1 gene was found in AD patients [51]. Moreover, recent studies reported an alteration of histone acetylation profiles in AD brains [52,53]. Epigenetic evidence suggests that AD-related dementia manifests after a progressive change in key cellular pathways, that gradually leads to brain dysfunction and neurodegeneration [54]. Metabolic stress occurring during critical phases of brain development may epigenetically program gene expression during the entire life and influence the vulnerability to age-related neurodegeneration [55]. However, how nutrient-related signals can lastingly influence brain health and modulate the risk of AD remains largely unexplored. Our findings reveal novel HFD-dependent transcription factor alterations and epigenetic changes directly linked to the expression of enzymes controlling APP metabolism and Aβ accumulation in the hippocampus. This evidence raises the question of which therapeutic approach may prevent or revert this epigenetic imprinting in the offspring. Maternal diet supplementation with omega-3 fatty acid or choline has been reported to ameliorate cognitive functions in both rats and humans [56,57]. It is also conceivable, but needs to be proven, that the supplementation of nutrients with epigenetic activity, such as folate, docosahexaenoic acid or short-chain fatty acids, during the early life stress might counteract the detrimental effects of maternal HFD on neurodegeneration, such as observed for cognitive deficits, anxiety behavior and hepatic damage [58,59,60]. In addition to the mother’s diet, the lifestyle of the offspring can also represent a therapeutic option by mitigating the harmful effects of maternal diet. For instance, the maternal or offspring’s high-fiber diet have been shown to attenuate cognitive and social deficits induced by maternal obesity [61]. Finally, we demonstrated that the exposure of the offspring to novel enriched environment including free access to a running-wheel reverted maternal HFD-induced epigenetic changes on the regulatory sequences of BDNF gene [9]. Studies on experimental models may provide novel target and dietary strategies to counteract the onset and progression of AD [62]. Recent evidence described how the therapeutic effect of adamantine-based nanoparticles on an AD rat model was elicited by the modulation of both STAT3 and NF-kB transcriptional activity [63]. Future studies are necessary to identify the relative weight of individual nutrients in the development of neurodegeneration, to understand if epigenetic biomarkers can contribute for the early detection of AD and to demonstrate whether epigenetic modulators are able to prevent or reverse neurodegeneration and dementia.

## Data Availability

The data presented in this study are available in Supplementary Table S1.

## Supplementary Materials

The following supporting information can be downloaded at: https://www.mdpi.com/article/10.3390/cells12020220/s1, Table S1: Gene regulation; Table S2: Antibodies; Table S3: Primers used for chip analyses.
