# Supplementary material for: Maternal High Fat Diet Anticipates the AD-like Phenotype in 3xTg-AD Mice by Epigenetic Dysregulation of Aβ Metabolism
Source: Cells. 2023 Jan 4;12(2):220. doi: 10.3390/cells12020220 (PMC9856666; doi:10.3390/cells12020220)
Supplement: Supplementary file 1 [file cells-12-00220-s001.zip › Supplementary Matherials.pdf]

Supplementary Table S1. GENE REGULATION

Refseq	Symbol	Description	Fold Change	*p*-Value
NM_175628	A2m	Alpha-2-macroglobulin	1.59	0.523688
NM_013454	Abca1	ATP-binding cassette, sub-family A (ABC1), member 1	1.12	0.745455
NM_009599	Ache	Acetylcholinesterase	1.26	0.646704
NM_007404	Adam9	A disintegrin and metallopeptidase domain 9 (meltrin gamma)	1.34	0.424105
NM_177034	Apba1	Amyloid beta (A4) precursor protein binding, family A, member 1	1.33	0.430311
NM_018758	Apba3	Amyloid beta (A4) precursor protein-binding, family A, member 3	1.33	0.261720
NM_009685	Apbb1	Amyloid beta (A4) precursor protein-binding, family B, member 1	1.15	0.860086
NM_009686	Apbb2	Amyloid beta (A4) precursor protein-binding, family B, member 2	1.33	0.547291
NM_146104	Aph1a	Anterior pharynx defective 1a homolog (C. elegans)	1.67	0.423375
NM_007467	Aplp1	Amyloid beta (A4) precursor-like protein 1	1.27	0.722291
NM_009691	Aplp2	Amyloid beta (A4) precursor-like protein 2	1.41	0.473123
NM_009692	Apoa1	Apolipoprotein A-I	1.67	0.306639
NM_009696	Apoe	Apolipoprotein E	0.84	0.595652
NM_007471	App	Amyloid beta (A4) precursor protein	0.71	0.839977
NM_011792	Bace1	Beta-site APP cleaving enzyme 1	**4.76**	**0.015838**
NM_019517	Bace2	Beta-site APP-cleaving enzyme 2	0.71	0.793287
NM_009738	Bche	Butyrylcholinesterase	0.99	0.982585
NM_007540	Bdnf	Brain derived neurotrophic factor	0.89	0.625365
NM_009810	Casp3	Caspase 3	0.99	0.991140
NM_007609	Casp4	Caspase 4, apoptosis-related cysteine peptidase	1.18	0.897619
NM_007659	Cdk1	Cyclin-dependent kinase 1	1.58	0.401086
NM_007668	Cdk5	Cyclin-dependent kinase 5	1.50	0.521562
NM_183294	Cdkl1	Cyclin-dependent kinase-like 1 (CDC2-related kinase)	1.12	0.930541
NM_009891	Chat	Choline acetyltransferase	1.77	0.089544
NM_013492	Clu	Clusterin	0.56	0.756672
NM_007798	Ctsb	Cathepsin B	1.33	0.962375
NM_009982	Ctsc	Cathepsin C	1.06	0.670115
NM_009983	Ctsd	Cathepsin D	1.58	0.308861
NM_007800	Ctsg	Cathepsin G	1.26	0.587461
NM_009984	Ctsl	Cathepsin L	1.13	0.622795
NM_177821	Ep300	E1A binding protein p300	1.35	0.777057
NM_023913	Ern1	Endoplasmic reticulum (ER) to nucleus signalling 1	**4.57**	**0.005916**
NM_008083	Gap43	Growth associated protein 43	1.06	0.995759

NM_010308	Gnao1	Guanine nucleotide binding protein, alpha o	0.79	0.947277
NM_010311	Gnaz	Guanine nucleotide binding protein, alpha z subunit	0.84	0.794213
NM_008142	Gnb1	Guanine nucleotide binding protein (G protein), beta 1	1.19	0.769099
NM_010312	Gnb2	Guanine nucleotide binding protein (G protein), beta 2	1.56	0.714193
NM_013531	Gnb4	Guanine nucleotide binding protein (G protein), beta 4	1.89	0.454148
NM_010313	Gnb5	Guanine nucleotide binding protein (G protein), beta 5	1.00	0.660407
NM_025277	Gng10	Guanine nucleotide binding protein (G protein), gamma 10	1.19	0.788950
NM_025331	Gng11	Guanine nucleotide binding protein (G protein), gamma 11	1.07	0.841063
NM_010316	Gng3	Guanine nucleotide binding protein (G protein), gamma 3	0.88	0.566693
NM_010317	Gng4	Guanine nucleotide binding protein (G protein), gamma 4	1.59	0.501457
NM_010318	Gng5	Guanine nucleotide binding protein (G protein), gamma 5	**2.24**	**0.036035**
NM_010319	Gng7	Guanine nucleotide binding protein (G protein), gamma 7	**3.36**	**0.007533**
NM_010320	Gng8	Guanine nucleotide binding protein (G protein), gamma 8	**2.36**	0.220630
NM_010314	Gngt1	Guanine nucleotide binding protein (G protein), gamma transducing activity polypeptide 1	0.93	0.940182
NM_023121	Gngt2	Guanine nucleotide binding protein (G protein), gamma transducing activity polypeptide 2	1.40	0.478175
NM_001031667	Gsk3a	Glycogen synthase kinase 3 alpha	0.71	0.849351
NM_019827	Gsk3b	Glycogen synthase kinase 3 beta	1.41	0.359260
NM_016763	Hsd17b10	Hydroxysteroid (17-beta) dehydrogenase 10	1.06	0.511186
NM_031156	Ide	Insulin degrading enzyme	**0.20**	**0.000970**
NM_010514	Igf2	Insulin-like growth factor 2	0.89	0.713865
NM_010554	Il1a	Interleukin 1 alpha	1.12	0.631255
NM_010568	Insr	Insulin receptor	0.85	0.496445
NM_008509	Lpl	Lipoprotein lipase	1.18	0.924373
NM_008512	Lrp1	Low density lipoprotein receptor-related protein 1	0.94	0.959813
NM_008514	Lrp6	Low density lipoprotein receptor-related protein 6	0.89	0.811740
NM_001080926	Lrp8	Low density lipoprotein receptor-related protein 8, apolipoprotein e receptor	1.61	0.383559
NM_010838	Mapt	Microtubule-associated protein tau	0.66	0.999831

NM_010824	Mpo	Myeloperoxidase	1.72	0.565523
NM_001039934	Map2	Microtubule-associated protein 2	0.70	0.720247
NM_144931	Nae1	NEDD8 activating enzyme E1 subunit 1	**2.11**	0.120261
NM_021607	Ncstn	Nicastrin	**0.19**	**0.023541**
NM_026361	Pkp4	Plakophilin 4	0.63	0.680570
NM_008872	Plat	Plasminogen activator, tissue	1.12	0.992679
NM_008873	Plau	Plasminogen activator, urokinase	1.59	0.232588
NM_008877	Plg	Plasminogen	1.56	0.375379
NM_011101	Prkca	Protein kinase C, alpha	0.75	0.984260
NM_008855	Prkcb	Protein kinase C, beta	1.06	0.655583
NM_011102	Prkcg	Protein kinase C, gamma	0.56	0.950422
NM_011103	Prkcd	Protein kinase C, delta	1.33	0.621815
NM_011104	Prkce	Protein kinase C, epsilon	1.53	0.303289
NM_008857	Prkci	Protein kinase C, iota	1.33	0.261720
NM_008859	Prkcq	Protein kinase C, theta	1.41	0.672387
NM_008860	Prkcz	Protein kinase C, zeta	0.79	0.723034
NM_008943	Psen1	Presenilin 1	1.00	0.690470
NM_011183	Psen2	Presenilin 2	1.12	0.498989
NM_008458	Serpina3c	Serine (or cysteine) peptidase inhibitor, clade A, member 3C	1.59	0.424057
NM_009221	Snca	Synuclein, alpha	**0.42**	0.989376
NM_033610	Sncb	Synuclein, beta	1.12	0.645409
NM_026842	Ubqln1	Ubiquilin 1	0.84	0.738966
NM_025407	Uqcrc1	Ubiquinol-cytochrome c reductase core protein 1	0.99	0.997635
NM_025899	Uqcrc2	Ubiquinol cytochrome c reductase core protein 2	0.94	0.958692
NM_007393	Actb	Actin, beta	0.67	0.849114
NM_009735	B2m	Beta-2 microglobulin	1.11	0.638706
NM_008084	Gapdh	Glyceraldehyde-3-phosphate dehydrogenase	1.00	N/A
NM_010368	Gusb	Glucuronidase, beta	1.26	0.612282
NM_008302	Hsp90ab1	Heat shock protein 90 alpha (cytosolic), class B member 1	1.08	0.590556

Supplementary Table S2. ANTIBODIES

Primary Antibody	Host	Catalogue Reference
pFoxO1^{ser256}	Rabbit	Cell Signaling Technology #9461
FoxO1	Rabbit	Cell Signaling Technology #2880
AKT	Rabbit	Cell Signaling Technology #9272
pAKT^{ser473}	Rabbit	Cell Signaling Technology #4060
pFox03a^{ser253}	Rabbit	Cell Signaling Technology #9466
FoxO3a	Rabbit	Cell Signaling Technology #2497
pCREB^{ser133}	Rabbit	Cell Signaling Technology #9198
CREB	Rabbit	Cell Signaling Technology #4820
pGSK3β^{ser9}	Rabbit	Cell Signaling Technology #9336
GSK3β	Rabbit	Cell Signaling Technology #12456
Actin	Rabbit	ABCAM ab8227
pSTAT5^{Tyr694}	Mouse	Cell Signaling Technology #9356
STAT5	Rabbit	Cell Signaling Technology #94205
pSTAT3^{Tyr705}	Rabbit	Cell Signaling Technology #9145
STAT3	Rabbit	Cell Signaling Technology #9139
pNF-kB^{Ser536}	Rabbit	ABCAM ab76302
NF-kB p65	Rabbit	ABCAM ab16502

Supplementary Table S3. PRIMERS USED FOR CHIP ANALYSES

Gene	Primer Sequences
BACE1	**FW1** TACTCAGCACAGGCCTTACC
	**REV1** AACTACATGGTCTACGTGTTCC
BACE1	**FW2** GAGAAGTGTATGATATTGGTTCC
	**REV2** CTAGGCAGGCTGGGGAGGC
ERN1	**FW1** AACACTTCAAATTGTGTCTGCTGC
	**REV1** GTTTTTGAAGAGAAAAGGTTATCC
ERN1	**FW2** GAGGAACGGATGGCTGCTGG
	**REV2** GACCTCTAGAGCAGAGCAAGG
IDE	**FW** TCCTTGCTTCCGTAGTCCACG
	**REV** GTCCTTCCTAGGCCAGCAGG
NICASTRIN	**FW** GGAGAGGCAAGATGGCTACG
	**REV** GCAGGTCCAGGCAAAGGAGG
