# Supplementary figures and images for: Maternal High Fat Diet Anticipates the AD-like Phenotype in 3xTg-AD Mice by Epigenetic Dysregulation of Aβ Metabolism
Source: Cells. 2023 Jan 4;12(2):220. doi: 10.3390/cells12020220 (PMC9856666; doi:10.3390/cells12020220)

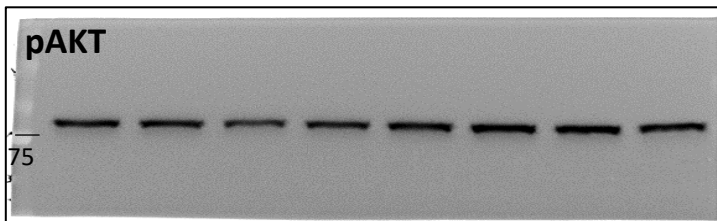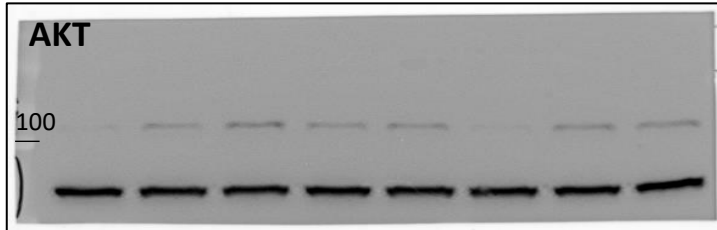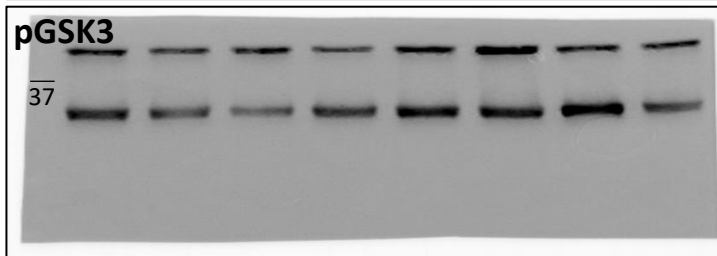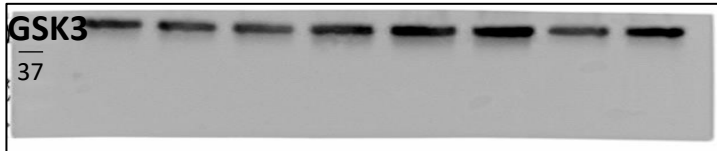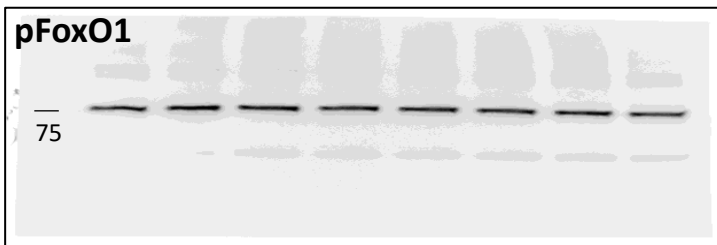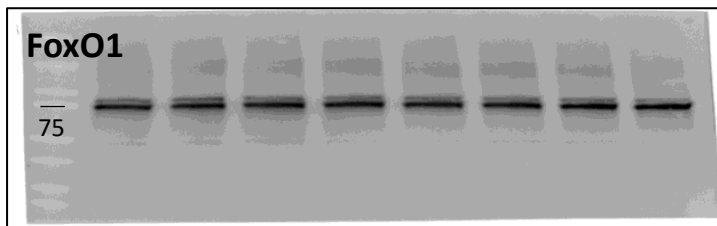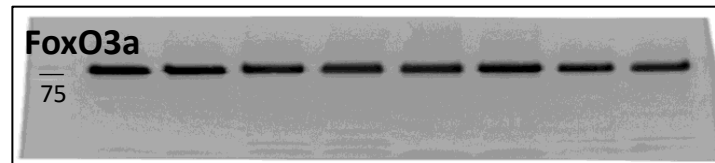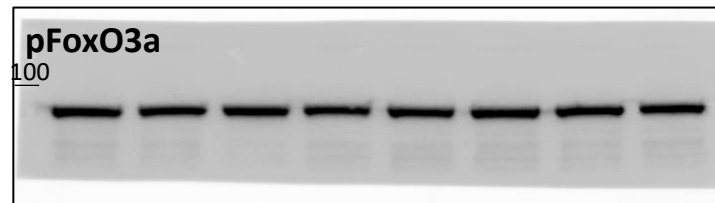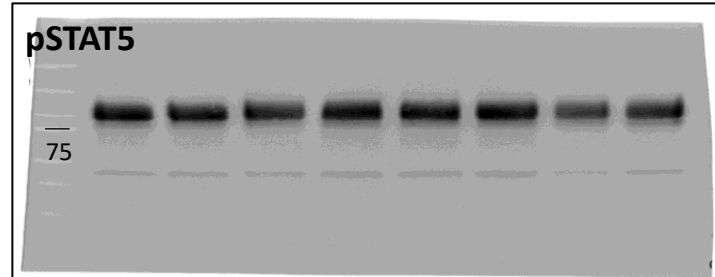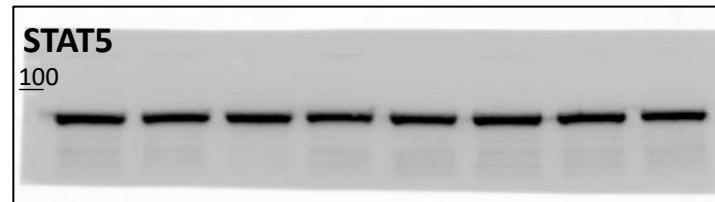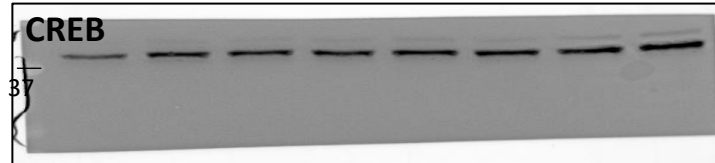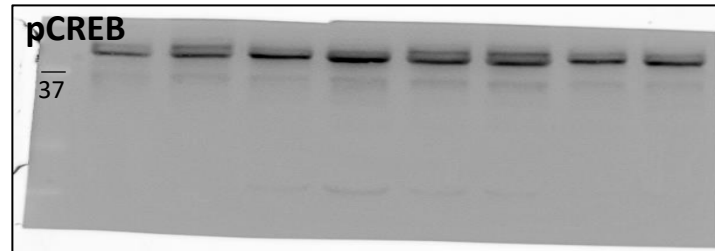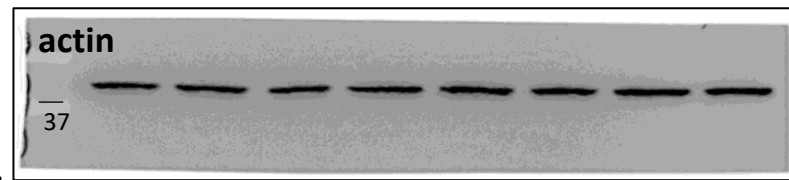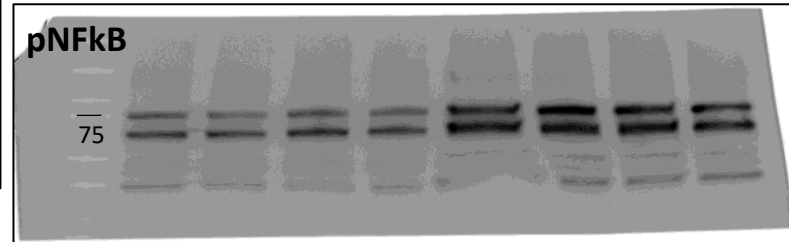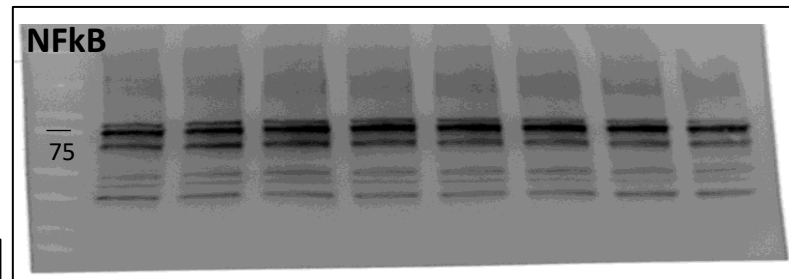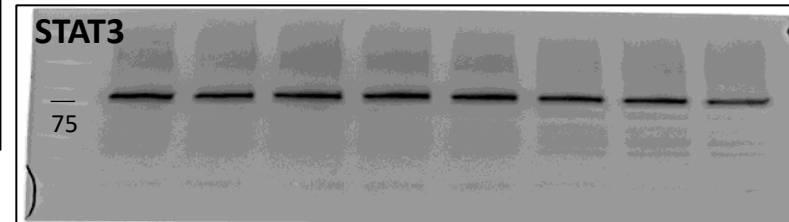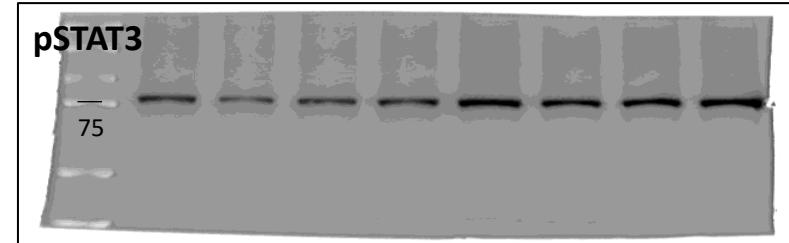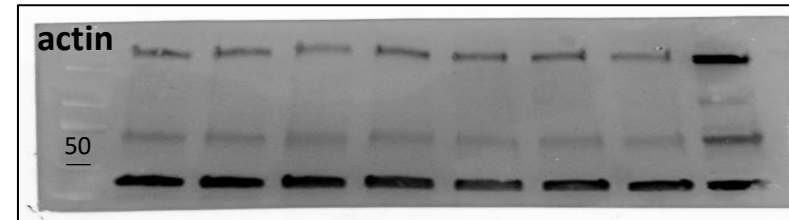

Supplement: Supplementary file 1 [file cells-12-00220-s001.zip › uncropped blots.pdf]
